# Ablation of *Enpp6* Results in Transient Bone Hypomineralization

**DOI:** 10.1002/jbm4.10439

**Published:** 2020-12-08

**Authors:** Scott Dillon, Karla Suchacki, Shun‐Neng Hsu, Louise A Stephen, Rongling Wang, William P Cawthorn, Alan J Stewart, Fabio Nudelman, Nicholas M Morton, Colin Farquharson

**Affiliations:** ^1^ The Roslin Institute and Royal (Dick) School of Veterinary Studies University of Edinburgh Midlothian UK; ^2^ Centre for Cardiovascular Science, Queen's Medical Research Institute University of Edinburgh Edinburgh UK; ^3^ School of Medicine University of St Andrews St. Andrews UK; ^4^ School of Chemistry University of Edinburgh Edinburgh UK

**Keywords:** ECTONUCLEOTIDE PYROPHOSPHATASE/PHOSPHODIESTERASE 6, MATRIX MINERALIZATION, MATRIX VESICLE, ORPHAN PHOSPHATASE 1, OSTEOBLAST

## Abstract

Biomineralization is a fundamental process key to the development of the skeleton. The phosphatase orphan phosphatase 1 (PHOSPHO1), which likely functions within extracellular matrix vesicles, has emerged as a critical regulator of biomineralization. However, the biochemical pathways that generate intravesicular PHOSPHO1 substrates are currently unknown. We hypothesized that the enzyme ectonucleotide pyrophosphatase/phosphodiesterase 6 (ENPP6) is an upstream source of the PHOSPHO1 substrate. To test this, we characterized skeletal phenotypes of mice homozygous for a targeted deletion of *Enpp6* (*Enpp6* ^*−/−*^). Micro‐computed tomography of the trabecular compartment revealed transient hypomineralization in *Enpp6* ^*−/−*^ tibias (*p* < 0.05) that normalized by 12 weeks of age. Whole‐bone cortical analysis also revealed significantly hypomineralized proximal bone in 4‐ but not 12‐week‐old *Enpp6* ^*−/−*^ mice (*p* < 0.05) compared with WT animals. Back‐scattered SEM revealed a failure in 4‐week‐old trabecular bone of mineralization foci to propagate. Static histomorphometry revealed increased osteoid volume (*p* > 0.01) and osteoid surface (*p* < 0.05), which recovered by 12 weeks but was not accompanied by changes in osteoblast or osteoclast number. This study is the first to characterize the skeletal phenotype of *Enpp6* ^*−/−*^ mice, revealing transient hypomineralization in young animals compared with WT controls. These data suggest that ENPP6 is important for bone mineralization and may function upstream of PHOSPHO1 as a novel means of generating its substrates inside matrix vesicles. © 2020 The Authors. *JBMR Plus* published by Wiley Periodicals LLC. on behalf of American Society for Bone and Mineral Research.

## Introduction

Biomineralization of the skeleton is a fundamental process indispensable for health and well‐being throughout life. The vertebrate skeleton is a hugely complex organ that performs varied and diverse functions encompassing its action as a biomechanical and protective scaffold in conjunction with the musculature, its role in calcium (Ca^2+^) ion and inorganic phosphate (P_i_) homeostasis, and recent evidence demonstrating its capacity as an endocrine organ involved with energy homeostasis.^(^ ^1^ ^)^ Matrix vesicles (MVs) are membrane‐bound nanospherical bodies 100 to 300 nm in diameter, which are associated with both physiological and pathological biomineralization.^(^ ^2^, ^3^ ^)^ Many in vitro and in vivo studies have shown the first mineral crystals in diverse mineralized tissues, such as bone, dentin, and cartilage, are affiliated with these vesicles in the extracellular matrix.^(^ ^4^, ^5^, ^6^ ^)^ MVs are hypothesized to facilitate regulated biomineralization through provision of a locally protected environment within which Ca^2+^ and P_i_ may accumulate in an amorphous state but that also enable transport and targeting of cargo to the preformed collagenous matrix.

The biochemical mechanisms through which P_i_ is generated within MVs are currently unclear. Orphan phosphatase 1 (PHOSPHO1; *Phospho1*) was identified as a novel bone phosphatase critical for mineralization.^(^ ^7^, ^8^, ^9^ ^)^ The PHOSPHO1 KO mouse (*Phospho1* ^*−/−*^) displays a hypomineralized skeleton at birth, biomechanical incompetency, scoliosis, and spontaneous greenstick fractures.^(^ ^10^, ^11^ ^)^ A key observation was the capacity of PHOSPHO1 to generate P_i_, with specific activity towards phosphocholine (PCho) and phosphoethanolamine (PEA).^(^ ^12^, ^13^, ^14^ ^)^ Although we and others have validated the presence of biologically active PHOSPHO1 within MVs,^(^ ^13^, ^15^ ^)^ how PEA and PCho are generated within the vesicles for hydrolysis by PHOSPHO1 is as yet unknown.

The current leading model for intravesicular PHOSPHO1 function involves the chemical transformation of vesicle membrane constituent phospholipids into appropriate enzymatic substrates for PHOSPHO1.^(^ ^3^, ^16^ ^)^ An as‐yet unidentified enzyme (or group of enzymes) with phospholipase A_2_ activity is first hypothesized to convert membrane‐constituent phosphatidylcholine to lysophosphatidylcholine.^(^ ^9^, ^16^ ^)^ Several vertebrate PLA_2_ isoforms are known to be expressed by chondrocytes and osteoblasts and were localized to actively mineralizing matrix in cartilage and metaphyseal bone.^(^ ^17^, ^18^ ^)^ However, specific candidates that may function as part of the MV mechanism have yet to be identified. Ectonucleotide pyrophosphatase/phosphodiesterase 6 (ENPP6; or glycerophosphocholine cholinephosphodiesterase) is a member of the nucleotide pyrophosphatase/phosphodiesterase family with lysophospholipase C activity, catalyzing the conversion of lysophosphatidylcholine to PCho with a monoacylglycerol byproduct.^(^ ^19^, ^20^, ^21^, ^22^ ^)^ Therefore, ENPP6 may act upstream of PHOSPHO1 to generate intravesicular PCho; however, the role of ENPP6 in bone mineralization has yet to be investigated. This study aims to characterize the skeletal phenotype of the global ENPP6 KO mouse to give insight into its role in bone formation.

## Materials and Methods

All chemical reagents were purchased from Sigma‐Aldrich (Gillingham, Dorset, UK) unless otherwise stated.

### Animals

All experimental protocols were approved after review by the University of Edinburgh Bioresearch and Veterinary Services and maintained in accordance with UK Home Office guidelines for the care and use of laboratory animals. Animals heterozygous for a targeted deletion of exon1 of *Enpp6* (*Enpp6* ^*+/−*^) were obtained from Taconic Biosciences (Rensselaer, NY, USA). The animal model repository was generated through ES‐cells of the 129/SvEv and C57BL/6 strain and subsequently maintained with B6129F1 mice. Colonies of WT (*Enpp6* ^*+/+*^) and KO (*Enpp6* ^*−/−*^) mice were created by crossing heterozygous animals and subsequently maintained as separate colonies. Mice were housed in standard polypropylene cages with *ad libitum* access to food and water and environmental enrichment. The facility maintained a 12‐hour light/dark cycle, and temperature was controlled at 19 to 22°C.

For skeletal phenotyping, female mice at 4 and 12 weeks of age were used. Animals were culled using Schedule 1 methods, and tissues were dissected for analysis. Tissues were processed according to the protocols detailed below for downstream analyses.

### Primary osteoblast cell culture

Calvaria were dissected from entire litters of 3‐ to 5 day‐old *Enpp6* ^*+/+*^ mice, and primary osteoblasts extracted using standard protocols.^(^ ^23^ ^)^ Isolated cells were cultured in αMEM supplemented with 10% FBS and 50‐μg/mL gentamicin (Invitrogen, Carlsbad, CA, USA) at 37°C in a humidified 5% CO_2_ atmosphere until 80% confluent, dissociated and seeded in 6‐well plates at 100,000 cells per well. Once 80% confluence was reached, cells were stimulated to assume an osteogenic phenotype, though media supplementation with 50 μg/mL L‐ascorbic acid and 1.5mM CaCl_2_ without an additional exogenous source of P_i_ as previously described.^(^ ^24^ ^)^

### Quantitative polymerase chain reaction

For RNA extraction from cells in culture, media was discarded, and cells were washed three times in warm Hank's balanced salt solution. Cell monolayers were scraped in 1‐mL cold Qiazol (QIAGEN, Hilden, Germany). Samples were snap frozen in liquid nitrogen and maintained at ‐70°C before processing. For RNA extraction from tissues, left femora were dissected, immediately snap frozen in liquid nitrogen, and maintained at ‐70°C before tissue processing. Samples were homogenized in 1 mL of cold Qiazol using an IKA T10 ULTRA‐TURRAX tissue homogenizer (IKA, Staufen, Germany).

RNA was isolated from both cell and tissue samples using the RNeasy Mini kit according to manufacturer's instructions (QIAGEN). RNA concentration was measured using nanodrop spectrophotometry (Thermo Fisher Scientific, Loughborough, UK), and purity assessed using the 260:280‐nm wavelength ratio. RNA was reverse transcribed using SuperScript II (Thermo Fisher Scientific, Waltham, MA, USA) as per the manufacturer's instructions. Gene expression was assayed using PrimerDesign PrecisionPlus Master Mix with premixed SYBR Green (PrimerDesign, Chandler's Ford, UK), using a Stratagene Mx3000P real‐time qPCR system (Agilent Technologies, Cheadle, UK). Gene expression data were normalized to *18S* and analyzed using the ΔΔCt method.^(^ ^25^ ^)^ Oligonucleotide primers were obtained from Sigma‐Aldrich (Supplementary Table S1).

### Immunohistochemistry and immunofluorescence

Right tibias were fixed in 4% paraformaldehyde (PFA) in PBS for 24 hours at 4°C with gentle agitation and stored in 70% ethanol. Tibias were decalcified in 10% EDTA, pH 7.4 for 14 to 21 days under agitation, with the solution changed every 2 to 3 days. Samples were washed in PBS, sectioned in the sagittal plane, processed to paraffin wax using a Leica ASP300S tissue processor (Leica Biosystems, Wetzlar, Germany) and embedded in paraffin blocks on the medial cut surface. Using a rotary microtome, 3‐μm sections were cut.

Deparaffinized slides were subject to enzyme‐mediated antigen retrieval through incubation in 1‐mg/mL trypsin in 0.1% CaCl_2_ at 37°C for 30 minutes. Endogenous peroxidases were quenched through incubation in 3% H_2_O_2_ in methanol for 30 minutes at room temperature. The Vectastain Elite ABC kit (Vector Laboratories, Upper Heyford, UK) was used according to the manufacturer's instructions with a rabbit anti‐ENPP6 primary antibody (ab224564; Abcam, Cambridge, UK) at a 1:20 dilution. Rabbit IgG was used at the same final concentration as the primary antibody in negative isotype controls.

For immunofluorescence, slides were blocked in 10% normal goat serum in 1% BSA for 1 hour at room temperature subsequent to antigen retrieval as above. Primary antibodies against ENPP6 (as above), tissue nonspecific alkaline phosphatase (TNAP; MAB2909; 1:500; R&D Systems, Minneapolis, MN, USA) and PHOSPHO1 (HCA093; 1:500; Bio‐Rad Laboratories, Deeside, UK) were diluted in 1% BSA and incubated on sections at 4°C overnight in a humidified environment. Negative control sections were incubated in buffer only. Anti‐rabbit AlexaFluor647 (A27040; Invitrogen), anti‐rat AlexaFluor488 (A‐11006; Invitrogen), and anti‐human AlexaFluor594 (A‐11014; Invitrogen) secondary antibodies were used at 2 μg/mL and incubated for 1 hour at room temperature. Slides were counterstained in Hoechst and mounted in ProLong Gold (Thermo Fisher Scientific, UK). Images were acquired using an LSM 880 laser scanning confocal microscope with a 63× oil‐immersion objective lens (Zeiss, Cambridge, UK).

### Micro‐computed tomography

For μCT, left tibias and phantom calibration standards were embedded in 1% agarose and scanned using a SkyScan 1172 desktop micro‐CT (Bruker, Kontich, Belgium) through 360 degrees in steps of 0.280 degrees with two‐frame averaging. A voxel resolution of 6.03 μm was obtained with the following settings; 54 kV source voltage, 185 μA source current, and an exposure time of 1767 ms, with a 0.5 mm aluminium filter. Data were reconstructed using NRecon v1.6.9 (Bruker). Reconstruction thresholding was optimized to encapsulate the target image, defined by attenuation coefficients of 0.005 to 0.100. Ring artifact correction was set to 5 and a beam‐hardening correction of 20% was applied. Consistent settings were used for all samples.

After reconstruction, CtAn v1.16.4 (Bruker) was used to analyze metaphyseal trabecular bone parameters as previously described.^(^ ^26^ ^)^ A 250‐slice subset of metaphyseal trabecular bone was used for analysis, using the base of the growth plate as a standard reference point. Whole‐bone cortical analysis was performed using the BoneJ image analysis package^(^ ^27^ ^)^ for Fiji.^(^ ^28^ ^)^ Reconstructed scans of individual bones were first consistently oriented using the moments of inertia function, the trabecular bone and fibula were removed from the images, and the data set cropped 10% to 90% or 15% to 90% of total bone length in 12‐ and 4‐week‐old bones, respectively, to exclude the epiphysis and proximal and distal growth plates. Cortical bone parameters were then analyzed using the BoneJ slice geometry function. Data were processed and visualized using R.^(^ ^29^ ^)^ BMD was calculated based on known‐density hydroxyapatite phantoms imaged under identical conditions as experimental samples, and appropriate calibration performed against attenuation coefficients in both CtAn and BoneJ.

### Back‐scattered scanning electron microscopy

Right humeri were fixed in 2.5% PFA; 2.5% glutaraldehyde in 0.1M sodium cacodylate overnight at 4°C with gentle agitation. Samples were bisected, dehydrated in an ethanol series, and subjected to CO_2_ critical point drying using a Polaron E3100 dryer (Quorum Technologies, East Sussex, UK). Humeri were embedded in an epoxy resin block, ground using 2500 grit silicon carbide paper with water until plane, and polished on a Metaserv polisher (Buehler, Lake Bluff, IL, USA) using 0.3‐μm aluminium oxide powder before a thin carbon coat was applied to the surface. The block was mounted on a 25‐mm stub and examined under back‐scattered SEM (BSE‐SEM) using a Sigma HD VP microscope (Zeiss) fitted with a four‐quadrant solid‐state angle selective backscatter detector and operating at 15 keV, at a working distance of 7 mm and aperture size of 30 μm. Energy‐dispersive X‐ray spectroscopy (EDS) was performed using the AZtecEnergy system (Oxford Instruments, Abingdon, UK).

### Three‐point bending

Left humeri were cleaned of soft tissue, frozen in distilled water and stored at ‐20°C. Three‐point bending was carried out using a Lloyd LRX5 materials testing machine (Lloyd Instruments, Bognor Regis, UK) fitted with a 100‐N load cell. The span was fixed at 10 mm and bones loaded with a cross‐head speed of 1 mm/min until failure as previously described.^(^ ^10^ ^)^

### Histology and static histomorphometrics

We stained 3‐μm sections of the right tibia (prepared as above) with H&E and Goldner's trichrome using standard protocols. For tartrate‐resistant acid phosphatae (TRAP) staining, 70‐mg napthol AS‐TR phosphate was dissolved in 250‐μL N‐N dimethyl formamide and added to 50 mL of 2.3‐mg/mL sodium tartrate dihydrate; 1.4 mg/mL fast red salt TR solution in 0.2M sodium acetate buffer (pH 5.2). Deparaffinized slides were incubated in staining solution at 37°C for 1 hour, counterstained in Meyer's hematoxylin, and mounted in aqueous mounting medium (Vector Laboratories). Slides were imaged using a NanoZoomer‐XR slide scanning system (Hamamatsu Photonics, Hamamatsu City, Shizuoka, Japan). Static histomorphometry was performed using the BIOQUANT OSTEO software package (BIOQUANT Image Analysis Corp, Nashville, TN, USA) as per the manufacturer's instructions and following ASBMR's bone histomorphometry guidelines.^(^ ^30^ ^)^

### Osmium bone marrow fat analysis

Following μCT analysis at 12 weeks, bones were removed from 1% agarose and washed in Sorensen's phosphate buffer (81mM KH_2_PO_4_, 19mM Na_2_HPO_4_ · 7H_2_O, pH 7.4) prior to decalcification. Tibia were decalcified in 14% EDTA for 2 weeks at 4°C, washed in Sorensen's phosphate buffer and stained with 1% osmium tetroxide solution (1% w/v; diluted 1:1 in Sorensen's phosphate buffer; Agar Scientific, Stansted, Essex, UK) for 48 hours at room temperature, washed, and stored in Sorensen's phosphate buffer at 4°C. Stained tibias were embedded in 1% agarose and scanned using a Skyscan 1172 desktop micro‐CT (Bruker) as previously described.^(^ ^31^, ^32^ ^)^ The data were reconstructed using Skyscan software NRecon v1.6.9.4 (Bruker). Volumetric analysis was performed using CTAn v1.13.5.1 (Bruker).

### Statistical analysis

Data are expressed as mean ± SD of at least four replicates per experiment. Sample size for each analysis is provided in the relevant figure legends. Significance testing was performed using either R^(^ ^29^ ^)^ or SPSS (IBM, Armonk, NY, USA) after appropriate testing for normality and homogeneity of variance. Statistical significance was defined by a *p* value of <0.05.

## Results

### ENPP6 is expressed in mature osteoblasts and is upregulated during mineralization in vitro

**Fig 1:**
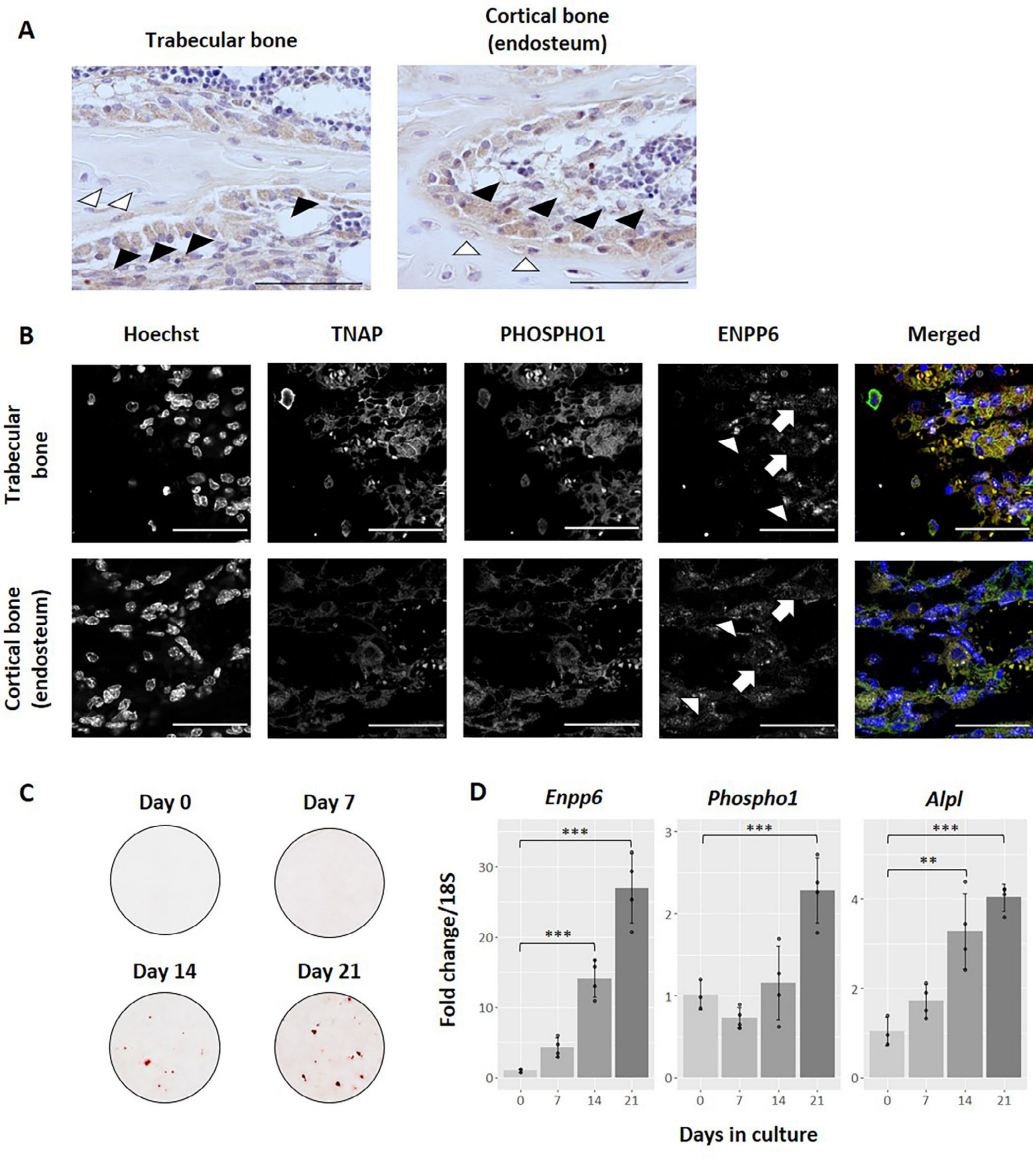
Ectonucleotide pyrophosphatase/phosphodiesterase 6 (ENPP6) is expressed at sites of bone formation and is upregulated during mineralization in vitro. (*A*) Immunohistochemistry using an anti‐ENPP6 primary antibody in 4‐week tibias shows expression in cells at mineralizing surfaces in both trabecular and cortical bone, including osteoblasts (black arrowheads) and early osteocytes (white arrowheads). Scale bars = 50 μm. (*B*) Immunofluoresence staining for ENPP6 in *Enpp6* ^*+/+*^ 4‐week tibias shows colocalization with mineralizing markers tissue nonspecific alkaline phosphatase (TNAP) and orphan phosphatase 1 (PHOSPHO1) in both trabecular and cortical bone. ENPP6 staining is evident in both nuclei (white arrowheads) and the cytoplasm (white arrows). Scale bar = 50 μm. (*C*) Alizarin red staining in primary osteoblasts showing mineralized nodules generated over a time course. (*D*) Gene expression analysis in primary osteoblasts shows upregulation of *Enpp6* over a mineralizing time course along with other mineralization markers including *Phospho1* and *Alpl*. *n* = 3 (day 0); *n* = 4 (day 7–day 21). **p > 0.01; ***p > 0.001.

The localization of ENPP6 was first investigated in *Enpp6* ^*+/+*^ tissue to ensure expression at the mineralizing front. Immunohistochemical staining in 4‐week WT tibias revealed expression localized strongly to osteoblasts at mineralizing surfaces in both trabecular and cortical bone (Fig. 1*A*; black arrowheads). Expression was also noted in early osteocytes at both sites (Fig. 1*A*; white arrowheads). Immunofluorescence staining with confocal microscopy revealed expression of ENPP6 in mature osteoblasts at trabecular and cortical sites, which also exhibited strong TNAP and PHOSPHO1 colocalization (Fig. 1*B*). Surprisingly, ENPP6 also showed strong nuclear (Fig. 1*B*; white arrowheads), along with cytoplasmic (Fig. 1*B*; white arrows) localization. Furthermore, primary osteoblasts exhibited a significant upregulation of *Enpp6* over a mineralizing time course in culture, along with both *Phospho1* and *Alpl* (*p* > 0.001; Fig. 1*C*,*D*).

### Ablation of *Enpp6* results in transient hypomineralization of trabecular and cortical bone and delayed propagation of mineralization foci

To elucidate differences in bone structure and/or mineral density in the absence of functional ENPP6, μCT of the left tibia was performed. The skeletal phenotype was hypothesized to recover over time in a similar manner to the *Phospho1* ^*−/−*^ phenotype^(^ ^9^ ^)^; therefore, animals were investigated at both 4 weeks and 12 weeks of age.

**Fig 2:**
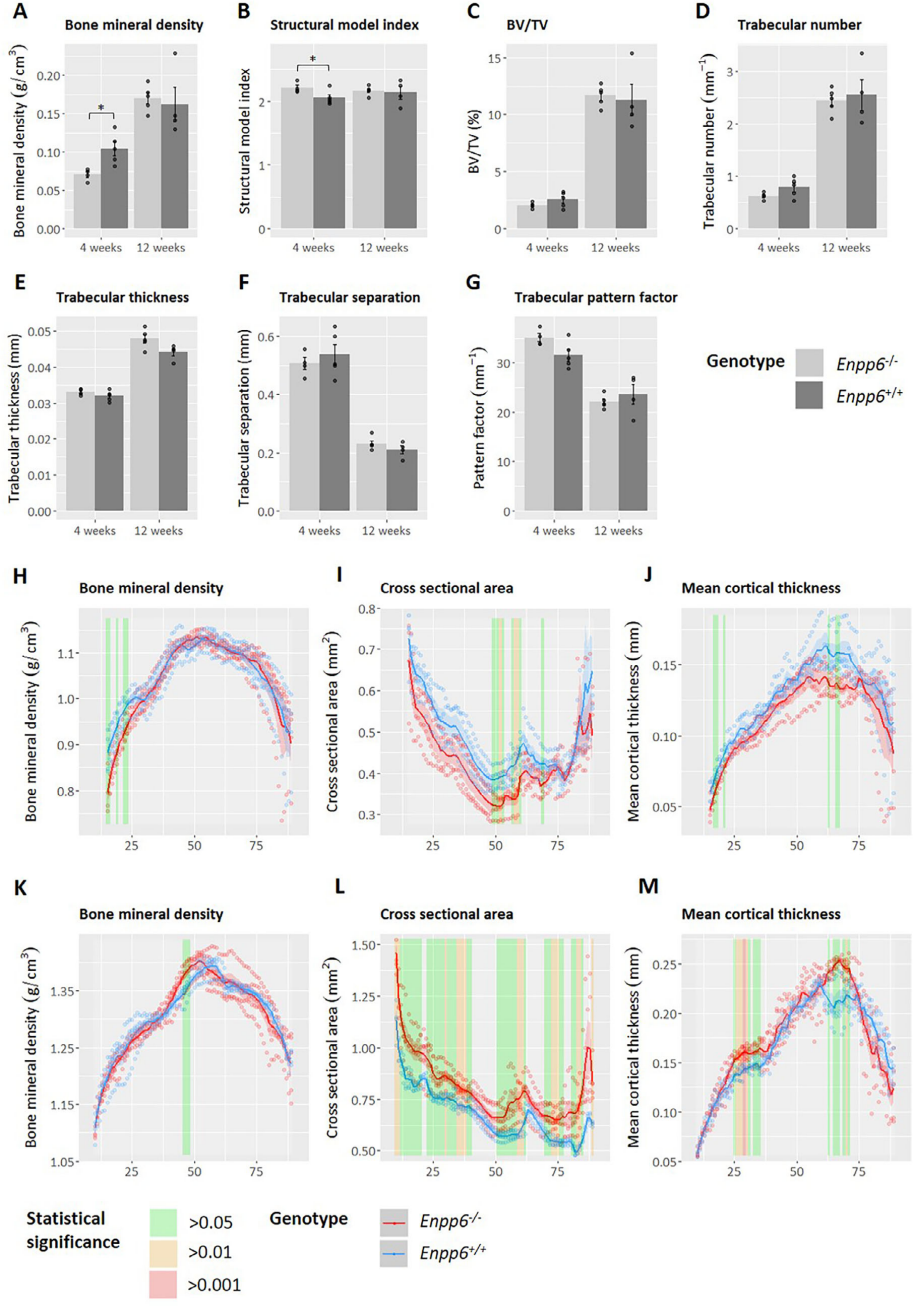
Micro‐computed tomography analysis of trabecular bone in *Enpp6* WT and KO tibias at 4 and 12 weeks of age shows hypomineralization of the trabecular and cortical bone compartment in 4‐week tibias and alteration of geometry. (*A*–*G*) Trabecular bone parameters measured from a 250‐slice volume distal to the growth plate. Data represent mean ± SD. *p > 0.05. (*H*–*M*) Whole‐bone cortical analysis at 4 weeks (*H*–*J*) and 12 weeks (*K*–*M*) from 15% and 10%, respectively, to 90% tibial length. Solid lines represent the mean ± SD. Individual data points are plotted within each % length bracket. The results of statistical significance testing between genotypes performed using multiple *t* tests with Bonferroni correction for multiple comparisons are reported at each length bracket using colored bars. BV/TV = Bone volume/total volume. *n* = 4‐week *Enpp6* ^*−/−*^ 4; 4‐week *Enpp6* ^*+/+*^ 5; 12‐week *Enpp6* ^*−/−*^ 5; *Enpp6* ^*+/+*^ 4.

At 4 weeks of age, trabecular bone at the proximal tibia was found to be significantly hypomineralized in *Enpp6* ^*−/−*^ compared with controls (*p* < 0.05; Fig. 2*A*). The structural model index (SMI) was also found to be significantly higher in *Enpp6* ^*−/−*^ samples (*p* < 0.05; Fig. 2*B*), with all other structural parameters showing no difference between genotypes (Fig. 2*C*–*G*). However, trabecular hypomineralization had recovered by 12 weeks of age, and SMI was similarly corrected (Fig. 2*A*,*B*). The remaining structural parameters remained consistent between genotypes at 12 weeks (Fig. 2*C*–*G*).

Whole‐bone cortical μCT analysis was conducted to investigate whether the hypomineralization observed in trabecular bone in young animals extended to the cortical bone compartment. Four‐week and 12‐week tibias were analyzed from 15% and 10% total length, respectively, to 90% total length to exclude the proximal and distal metaphyses and growth plates. Cortical bone in 4‐week *Enpp6* ^*−/−*^ animals was found to be hypomineralized with respect to controls in proximal regions (*p* < 0.05) but were mineralized normally more distally (Fig. 2*H*). Hypomineralized regions coincided with reductions in cross‐sectional area and mean cortical thickness; however, both were also significantly lower in other regions at the midshaft and more distally (Fig. 2*I*,*J*). Moments of inertia about the major and minor axes were also quantified to measure changes in cortical geometry. I_max_ and I_min_ were found to be significantly reduced in some midshaft‐distal regions (0.01 < *p* < 0.05; Supplementary Fig. S1 *A*,*B*).

The hypomineralized phenotype was found to be corrected in *Enpp6* ^*−/−*^ proximal cortical bone at 12 weeks. Surprisingly, a small significantly hypermineralized area was found at the midshaft in *Enpp6* ^*−/−*^ samples (*p* < 0.05; Fig. 2*K*). Furthermore, generalized increases throughout the length of 12‐week *Enpp6* ^*−/−*^ tibias were observed in cross‐sectional area, mean cortical thickness, and I_min_ and I_max_ (0.001 < *p* < 0.05; Fig. 2*L*,*M*; Supplementary Fig. S1 *C*,*D*).

**Fig 3:**
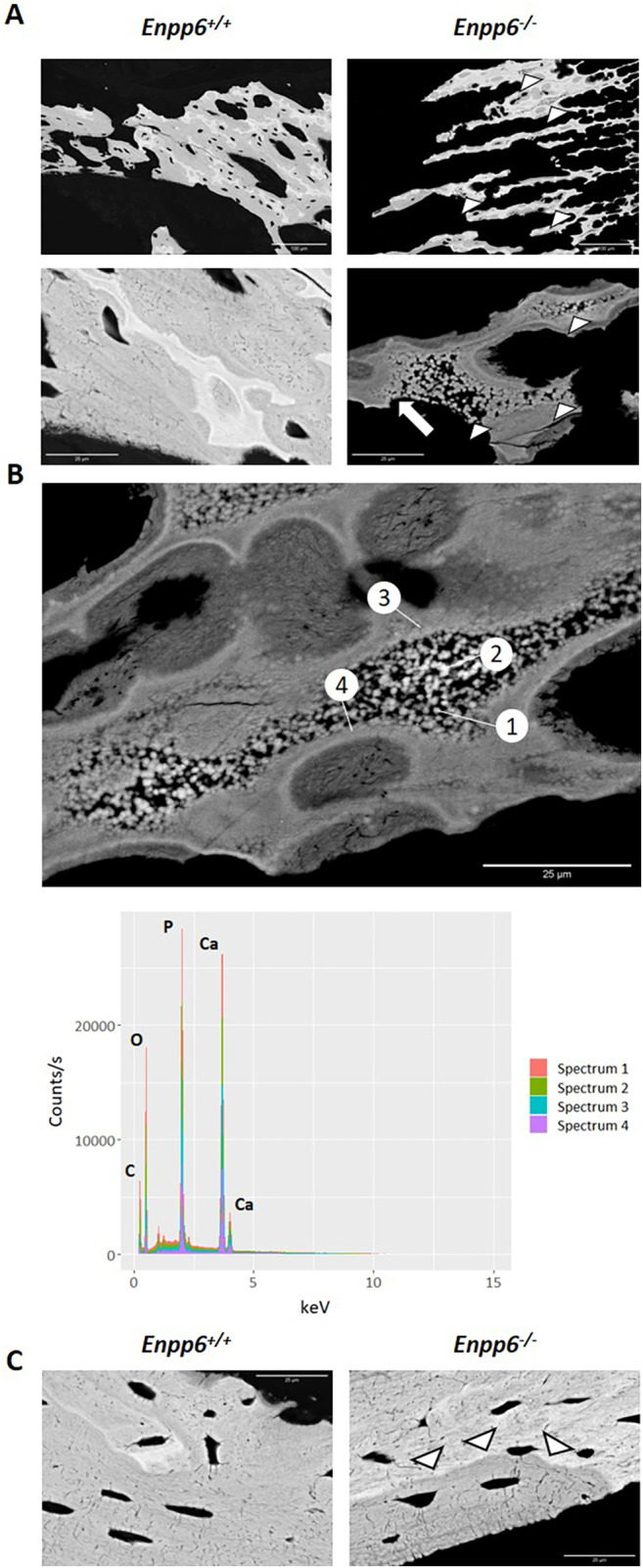
Back‐scattered SEM of *Enpp6* ^*+/+*^ and *Enpp6* ^*−/−*^ 4‐week humeri showing hypomineralization within both trabecular and cortical bone compartments. (*A*) Trabecular bone exhibits retention of large areas of calcified cartilage in *Enpp6* ^*−/−*^ animals and a failure of mineralization foci to propagate (white arrowheads). Foci fused with the surrounding developing trabecula (white arrow). (*B*) Energy‐dispersive spectroscopy of mineralization foci and surrounding tissue taken at the numbered positions. Spectra show enrichment of P, Ca, O, and C confirming electron‐dense particles represent mineralization foci. (*C*) Cortical bone at the endosteal surface also exhibits hypomineralization in *Enpp6* ^*−/−*^ bones (white arrowheads). P = Phosphorous; Ca = Calcium; O = Oxygen; C = Carbon. Scale bars = 25 μm.

Hypomineralization of both cortical and trabecular bone in 4‐week samples was confirmed by BSE‐SEM. In trabecular bone, large areas of retained calcified cartilage were evident in *Enpp6* ^*−/−*^ animals compared with the controls (Fig. 3*A*). Furthermore, several regions exhibited small (~1 μm) electron‐dense particles embedded within developing trabeculae, which were not present in control samples (Fig. 3*A*; white arrowheads). Particles progressively aggregated to fuse with the surrounding tissue (Fig. 3*A*; white arrow). The elemental composition of these particles was investigated by EDS revealing enrichment in calcium, phosphorus, oxygen, and carbon (Fig. 3*B*), with some other trace elements including sodium and magnesium (data not shown). Particles were generally similar in composition to the surrounding mineralized tissue (Fig. 3*B*). Therefore, particles likely represent mineralization foci that have failed to propagate in the absence of ENPP6. Generalized hypomineralization of diaphyseal cortical bone at the endosteal surface was also apparent in *Enpp6* ^*−/−*^ animals (Fig. 3*C*; white arrowheads).

### *Enpp6* ^*−/−*^ bones exhibit altered material properties but no change in bone cell activity

**Fig 4:**
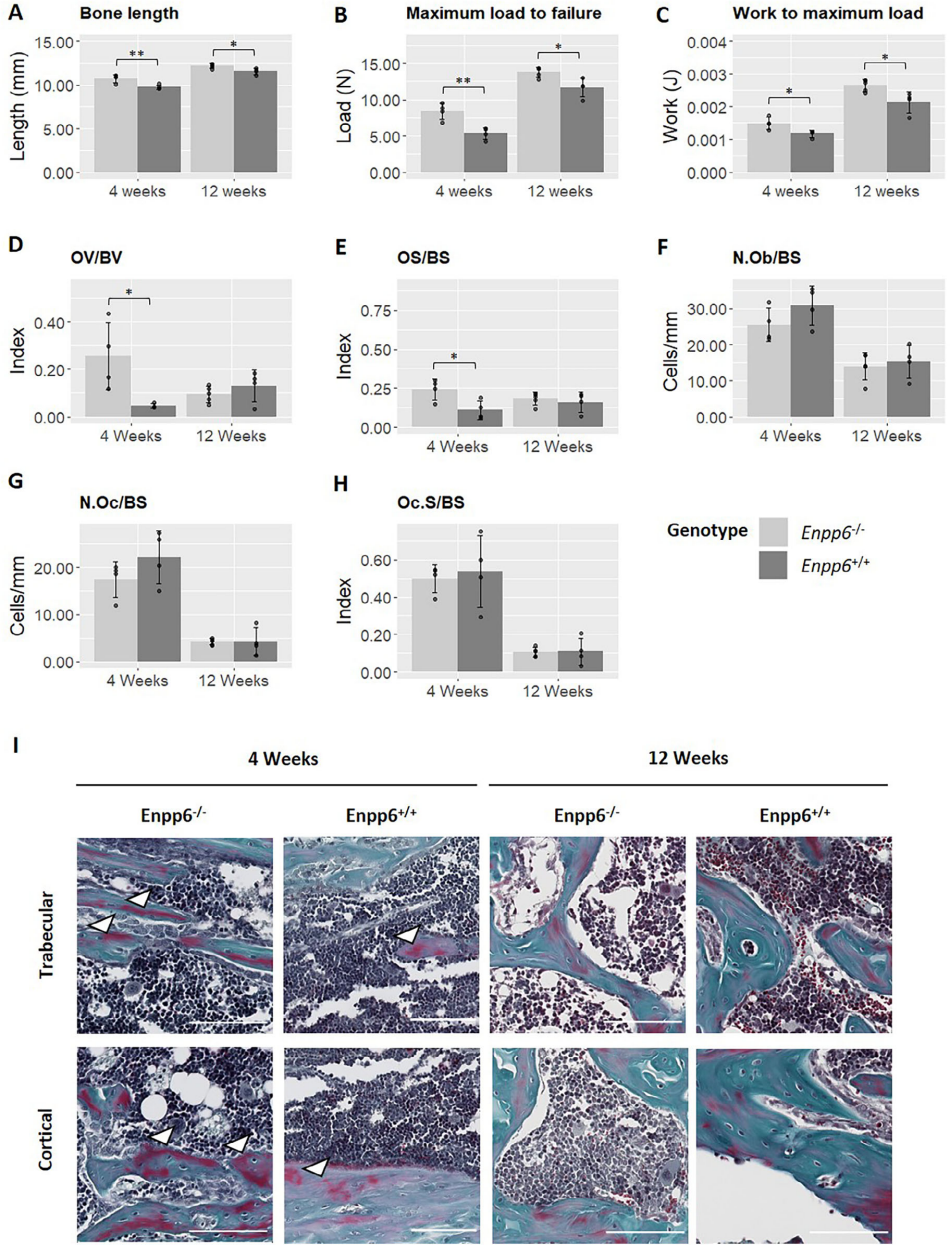
Mechanical testing and static histomorphometry show altered mechanical properties and hyperosteoidosis, without a change in bone cell activity. (*A*–*C*) three‐point bending in KO and WT humeri showing significantly altered mechanical properties of *Enpp6* ^*−/−*^ bones in (*A*) bone length; (*B*) maximum load to failure; and (*C*) work to maximum load. (*D*,*E*) Static histomorphometry within the trabecular compartment of 4‐ and 12‐week tibias shows hyperosteoidosis in young animals that subsequently recovers in older animals. (*F*–*H*) No differences in either age group were evident between genotypes in either normalized osteoblast or osteoclast number, or osteoclast surface. Data represent mean ± SD. OV/BV = osteoid volume/bone volume; OS/BS = osteoid surface/bone surface; N.Ob/BS = number of osteoblasts/bone surface; N.Oc/BS = number of osteoclasts/bone surface; Oc.S/BS = osteoclast surface/bone surface; *n* = 4‐week *Enpp6* ^*−/−*^ 4; 4‐week *Enpp6* ^*+/+*^ 4; 12‐week *Enpp6* ^*−/−*^ 4; *Enpp6* ^*+/+*^ 5. *p > 0.05; **p > 0.01.

Three‐point bending was performed to investigate whether observed changes between genotypes influences bone material properties. Both 4‐ and 12‐week *Enpp6* ^*−/−*^ humeri were significantly longer than WT counterparts (*p* < 0.01 and *p* < 0.05, respectively; Fig. 4*A*), although the average difference between genotypes was reduced in the older animals. Similarly, maximum load to failure was significantly higher at both 4 and 12 weeks (*p* < 0.01, *p* < 0.05; Fig. 4*B*) as was work to maximum load (*p* < 0.05; Fig. 4*C*).

Static histomorphometry within the trabecular compartment of the tibia was performed to determine whether hypomineralization resulted from altered bone cell activity. Significant increases in osteoid volume (*p* > 0.05) and osteoid surface (*p* > 0.05) were observed in 4‐week *Enpp6* ^*−/−*^ animals compared with controls, which had normalized by 12 weeks of age (Fig. 4*D*,*E*). This was not accompanied by an increase in osteoblast number (Fig. 4*F*). No differences were detected in either osteoclast number or surface between genotypes in either age group (Fig. 4*G*,*H*). Accumulation of large osteoid seams was evident in 4‐week *Enpp6* ^*−/−*^ tissue compared with controls, both in trabecular and cortical bone (Fig. 4*I*; white arrowheads), whereas differences had normalized by 12 weeks of age (Fig. 4*I*).

### Altered skeletal mineralization is not accompanied by changes in bone marrow adipose tissue volume

**Fig 5:**
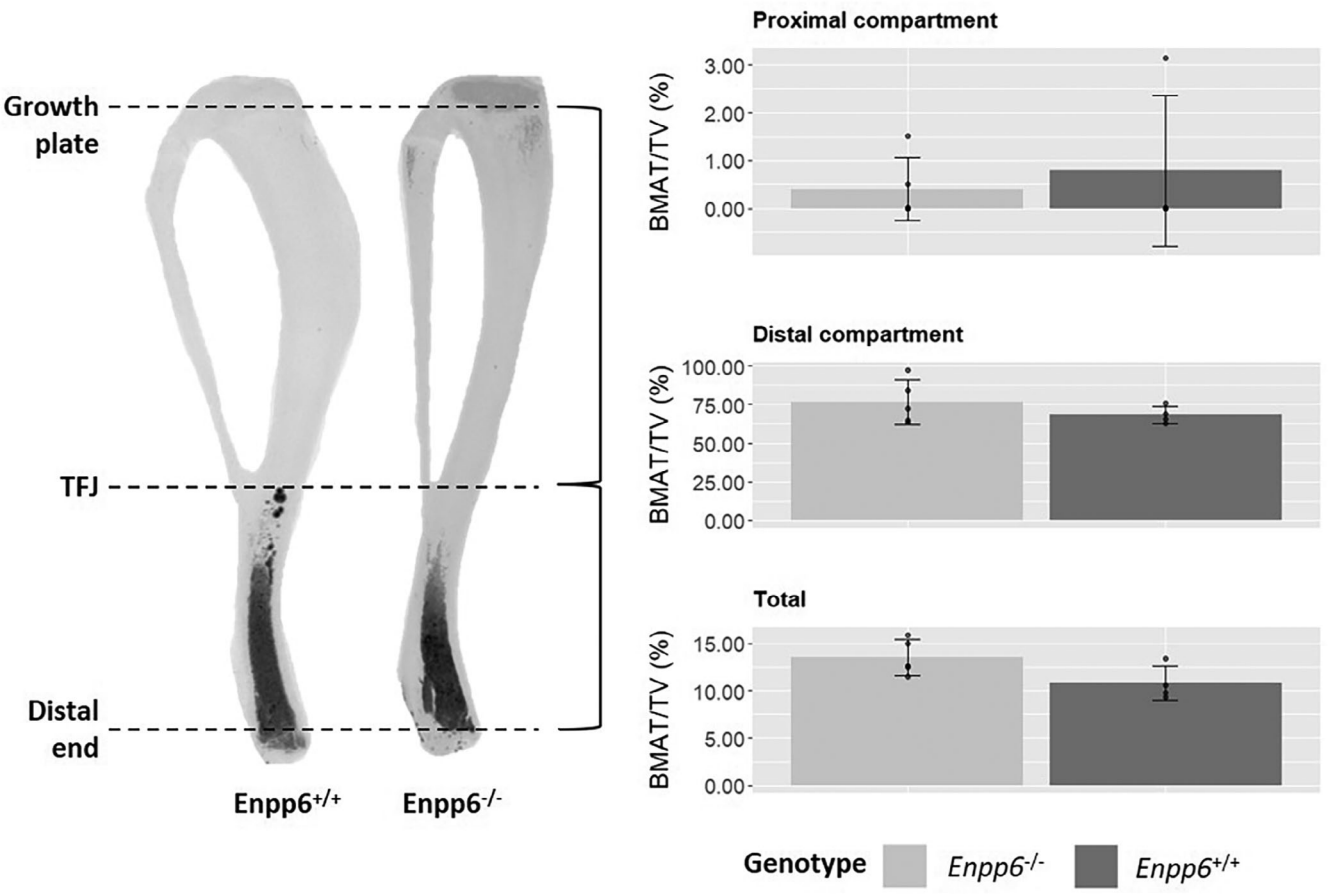
Quantitative bone marrow fat analysis shows no significant differences in the volume of bone marrow adipose tissue normalized to tissue volume within either the proximal or distal compartments of the tibia between genotypes in 12‐week animals. BMAT volume is expressed as % volume normalized to medullary cavity volume. The proximal medullary compartment was defined from the growth plate (G/P) to the tibiofibular joint (T/FJ) and distal compartment as from the T/FJ to the distal extremity of the medullary cavity. BMAT/TV = bone marrow adipose tissue/total volume. Data represent mean ± SD. *n* = 4 WT; 5 *Enpp6* ^*−/−*^. *n* = *Enpp6* ^*−/−*^ 5; *Enpp6* ^*+/+*^ 4.

As ENPP6 has also been implicated in fat metabolism, we investigated bone marrow adipose tissue (BMAT) volume in these mice.^(^ ^33^ ^)^ Recent evidence implicates a link between bone and energy metabolism, and we therefore hypothesized that ablation of *Enpp6* and induced hypomineralization would effect changes in BMAT.^(^ ^1^ ^)^ Osmium tetroxide staining and μCT analysis of 12‐week WT and *Enpp6* ^*−/−*^ tibia revealed that there was no alteration of BMAT volume despite hypomineralization in trabecular and cortical bone (Fig. 5). These analyses were not attempted in 4‐week bones as BMAT is not yet evident in these young animals.

## Discussion

This study is the first to characterize the skeletal phenotype of the *Enpp6* ^*−/−*^ mouse, revealing transient hypomineralization in young animals compared with controls, accompanied by impaired structural and functional properties and a failure of mineralization foci in the trabecular compartment to propagate and fuse. To date, the function of ENPP6 during bone mineralization has not received much attention; however, a few studies have provided incidental evidence to suggest it has a role to play. Upregulation of *Enpp6* expression has been found during osteogenesis, and therefore active bone mineralization in diverse contexts, including osseointegration of dental implants^(^ ^34^ ^)^ and enhanced bone modeling in response to a combination of sclerostin‐neutralizing antibody treatment and in vivo mechanical loading.^(^ ^35^ ^)^ Consistent with these observations, *Enpp6* expression is lower in bones of the Hyp mouse at 12 days of age (a murine homolog of X‐linked hypophosphatemic rickets) compared with controls.^(^ ^36^ ^)^ Downregulation was also observed in a CRISPR Cas9‐engineered osteogenesis imperfecta type V mouse model resulting in severe skeletal and mineralization defects in late‐stage mouse embryos, potentially caused by a deficiency in osteoblast differentiation.^(^ ^37^ ^)^ Furthermore, overexpression of Runx2 in mouse limb bud cultures leads to a more than threefold increase in Enpp6, which is well‐correlated with the upregulation observed in established mineralization markers including *Phospho1* and *Bglap2*, among others.^(^ ^38^ ^)^

Therefore, the expression of the *Enpp6* gene in osteogenic cells appears to be correlated with mineralization. Our observations agree with those from the literature, confirming ENPP6 localization to mineralizing cells including osteoblasts and early osteocytes. Furthermore, immunofluorescence staining co‐expresses with other mineralization markers such as TNAP and PHOSPHO1. Nuclear in addition to cytoplasmic localization of ENPP6 observed here may indicate that ENPP6 plays other roles in the cell in addition to its potential function in mineralization. The targeting mechanisms regulating ENPP6 are currently unclear, and previous work has shown distinct localizations of the protein in oligodendrocytes during brain development, for example.^(^ ^22^ ^)^ Taken together with in vitro results reported here showing an increase of *Enpp6* expression in primary calvarial osteoblasts over a mineralizing time course, these data provide evidence of a role for ENPP6 during bone formation.

The specific function of ENPP6 remains unclear, however, given its established lysophospholipase C activity.^(^ ^19^, ^20^, ^21^ ^)^ The similarities of the phenotype described here to that of the *Phospho1* ^*−/−*^ mouse are clear. As discussed above, ablation of PHOSPHO1 in mice induces hypomineralization in young animals, resulting in rickets/osteomalacia and biomechanical deficiency.^(^ ^9^, ^10^, ^11^ ^)^ Here we report that 4‐week *Enpp6* ^*−/−*^ tibias exhibit hypomineralized trabecular bone compared with controls, along with a significant increase in SMI indicating a change in trabecular geometry. Whole‐bone cortical analysis revealed the same trend with significantly reduced mineral density in proximal regions (ie, young tissue) and decreased mean cortical thickness, cross‐sectional area, and I_min_ at these and midshaft sites. At 12 weeks these differences not only recovered but in many areas along the length of the tibia exhibited significant increases in the KO compared with the WT, potentially indicating a compensatory response. Interestingly, this was not accompanied by any changes in expression of mineralization‐associated genes, including *Alpl* and *Phospho1*, which were upregulated on average at 12 weeks in *Enpp6* ^*−/−*^ bones but did not reach the level of statistical significance (Supplementary Fig. S2). Furthermore, we observed hyperosteoidosis in young animals that subsequently recovered (Fig. 4). The pattern of staining observed here is unusual, with some hypomineralized tissue lying deep to the bone surface. Although this pattern is curious, this correlates well with the BSE‐SEM data, revealing a failure of mineralization foci to propagate within some trabeculae in *Enpp6*‐null 4‐week animals. This may also correspond to the patchy osteomalacia previously described in mice lacking PHOSPHO1.^(^ ^11^ ^)^ We also report significant increases in maximum load to failure and work to maximum load in 4‐ and 12‐week *Enpp6* ^*−/−*^ humeri. Given the evident bone hypomineralization, this is a surprising finding but may represent an increased elasticity and plastic deformation under load as has also been shown in *Phospho1* ^*−/−*^ bones.^(^ ^10^ ^)^ It is also possible that altered material properties are a product of the structural changes in, for example, cortical thickness as shown here; further work is required to explicitly define this relationship.

The *Enpp6* ^*−/−*^ phenotype appears to represent an attenuated form of that evident in *Phospho1* ^*−/−*^ animals which recovers over time. Our data are consistent with the hypothesis that ENPP6 functions upstream of PHOSPHO1 to generate PCho and thereby facilitate generation of P_i_ for bone mineralization at the early stages of bone development. Several authors proposed this relationship, implicating the action of an as‐yet unidentified enzyme with phospholipase A_2_ activity and ENPP6 in combination upstream of PHOSPHO1 in scavenging P_i_ from the MV membrane.^(^ ^3^, ^16^ ^)^ Although a more direct pathway might be to generate PCho by hydrolysis of phosphatidylcholine (PC) through an enzyme with phospholipase C (PLC) activity, PC‐specific PLCs have yet to be found in mineralizing mammalian cells.^(^ ^17^ ^)^ During mineralization, the lipid composition of the MV membrane changes dramatically, showing the degradation of phospholipids such as PC and phosphatidylethanolamine (PE) and high concentrations of lysophospholipid breakdown products.^(^ ^39^, ^40^, ^41^, ^42^ ^)^ These studies establish that phospholipases, including those with lysophospholipase C activity such as ENPP6, are active within the MV and may function to provide substrates for PHOSPHO1 and other phosphatases. This mechanism would allow the intravesicular accumulation of P_i_ within a locally protected chemical environment, but also may function to progressively destabilize the vesicle membrane and facilitate release of its cargo.

The mineralization defects observed here in 4‐week *Enpp6* ^*−/−*^ animals may represent disruption of this mechanism, resulting in delayed phosphate accumulation and mineral nucleation. However, the milder nature of this phenotype (compared with *Phospho1* ^*−/−*^ mice) and its recovery by 12 weeks of age suggests other concurrent mechanisms are at play, but that ENPP6 plays a significant role during early postnatal bone development. PHOSPHO1 has been shown to hydrolyze PEA in addition to PCho.^(^ ^14^ ^)^ Ablation of ENPP6 and resulting loss of PCho accumulation may, therefore, only represent one pool of the substrate available for PHOSPHO1‐mediated P_i_ generation. The mechanism through which PEA may be generated inside MVs has yet to be established, however. In addition, sphingomyelin phosphodiesterase 3 (SMPD3; *Smpd3*) catalyzes hydrolysis of the phospholipid sphingomyelin to ceramide and PCho, and has also been implicated in this process.^(^ ^43^ ^)^ In a similar manner to PC and PE, sphingomyelin is also enriched within the MV membrane and undergoes degradation during mineralization.^(^ ^17^, ^39^, ^40^, ^41^, ^42^ ^)^ Furthermore, mutation of *Smpd3*, as in the fragilitas ossium (*fro**/**fro*) mouse model, causes severe skeletal defects, phenocopying noncollagenous osteogenesis imperfecta and resulting in bone hypomineralization and rickets/osteomlacia.^(^ ^44^, ^45^ ^)^ Although both ENPP6 and SMPD3 may generate PCho upstream of PHOSPHO1, it is also likely that this mechanism works synergistically with the established function of TNAP. The critical cooperation between PHOSPHO1 and TNAP can be seen dramatically in the double KO *Phospho1* ^*−/−*^ *;Alpl* ^*−/−*^ mouse, which exhibits a complete lack of skeletal mineralization and perinatal lethality.^(^ ^46^ ^)^

This research has for the first time highlighted a significant role for ENPP6 in bone formation and mineralization. These data provide a promising avenue of investigation; further research is required to confirm ENPP6 activity as part of the PHOSPHO1 pathway during MV‐mediated biomineralization.

## Disclosure

All authors state that they have no conflicts of interest.

## AUTHOR CONTRIBUTIONS

**Scott Dillon:** Conceptualization; data curation; formal analysis; investigation; methodology; visualization; writing‐original draft; writing‐review and editing. **Karla Suchacki:** Formal analysis; investigation; methodology; resources; visualization; writing‐review and editing. **Shun‐Neng Hsu:** Investigation; writing‐review and editing. **Louise Stephen:** Investigation; writing‐review and editing. **Rongling Wang:** Resources; writing‐review and editing. **William Cawthorn:** Methodology; resources; writing‐review and editing. **Alan Stewart:** Conceptualization; writing‐review and editing. **Fabio Nudelman:** Funding acquisition; project administration; supervision; writing‐review and editing. **Nik Morton:** Funding acquisition; resources; writing‐review and editing. **Colin Farquharson:** Conceptualization; funding acquisition; project administration; supervision; writing‐review and editing.

### PEER REVIEW

The peer review history for this article is available at https://publons.com/publon/10.1002/jbm4.10439.

## Supporting information


**Supplementary Figure S1** (A) Isotype negative control using normal rabbit serum in place of the primary antibody during immunohistochemical staining, demonstrating no apparent non‐specific staining. (B) Secondary‐only negative control imaged under identical conditions as positive slides for immunofluorescence, demonstrating no apparent non‐specific staining. Scale bars = 50 μm.

**Supplementary Figure S2** Micro‐computed tomography whole‐bone cortical analysis of moments of inertia in (A,B) 4‐week and (C,D) 12‐week tibias measured from 15% and 10% respectively to 90% bone length. Solid lines represent the mean ± standard deviation. Individual data points are plotted within each % length bracket. The results of statistical significance testing between genotypes performed using multiple t‐tests with Bonferroni correction for multiple comparisons are reported at each length bracket using colored bars. n = 4‐week *Enpp6* ^*−/−*^ 4; 4‐week *Enpp6* ^*+/+*^ 5; 12‐week *Enpp6* ^*−/−*^ 5; *Enpp6* ^*+/+*^ 4.

**Supplementary Table S1** Oligonucleotide primers used in quantitative polymerase chain reaction experiments.
