# Supplementary figures and images for: Ablation of Enpp6 Results in Transient Bone Hypomineralization
Source: JBMR Plus. 2020 Dec 8;5(2):e10439. doi: 10.1002/jbm4.10439 (PMC7872340; doi:10.1002/jbm4.10439)

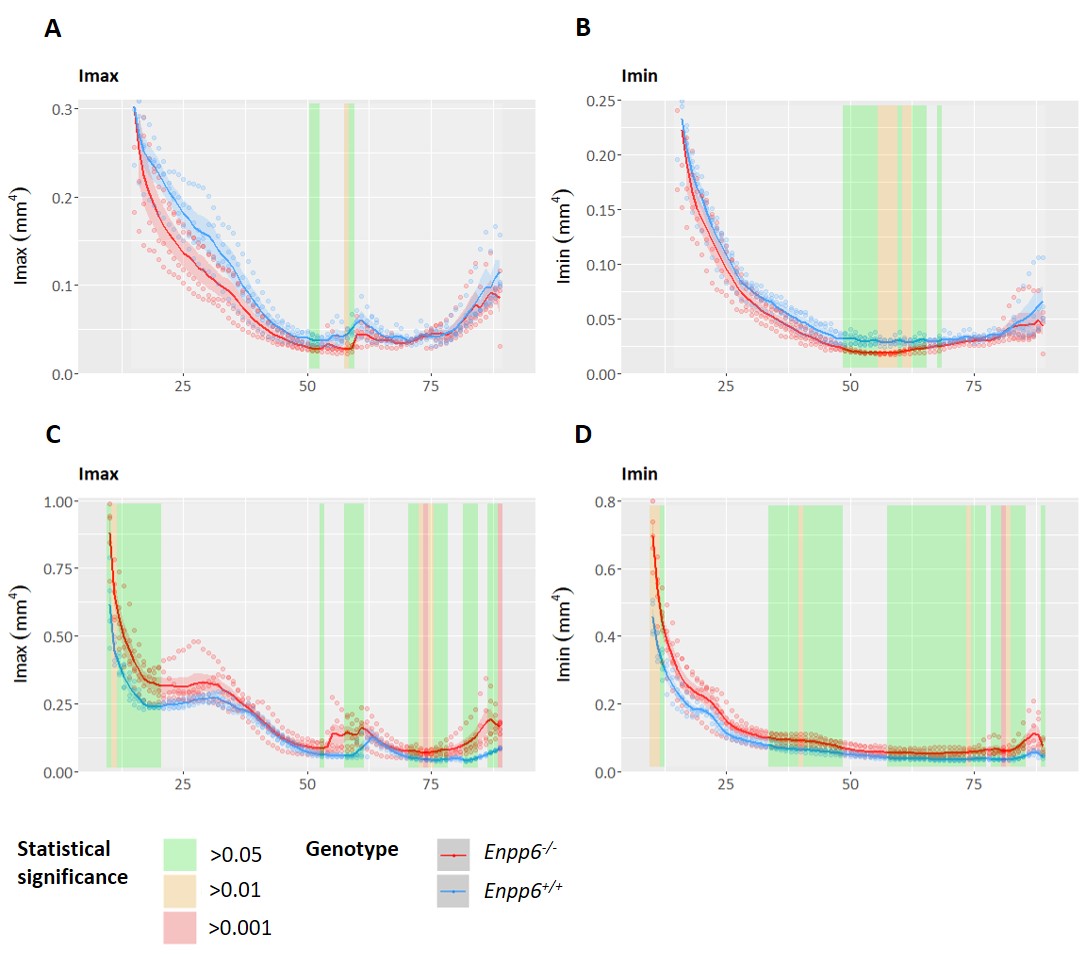

Supplement: Supplementary file 1 — Supplementary Figure S1 (A) Isotype negative control using normal rabbit serum in place of the primary antibody during immunohistochemical staining, demonstrating no apparent non‐specific staining. (B) Secondary‐only negative control imaged under identical conditions as positive slides for immunofluorescence, demonstrating no apparent non‐specific staining. Scale bars = 50 μm. [file JBM4-5-e10439-s001.jpg]

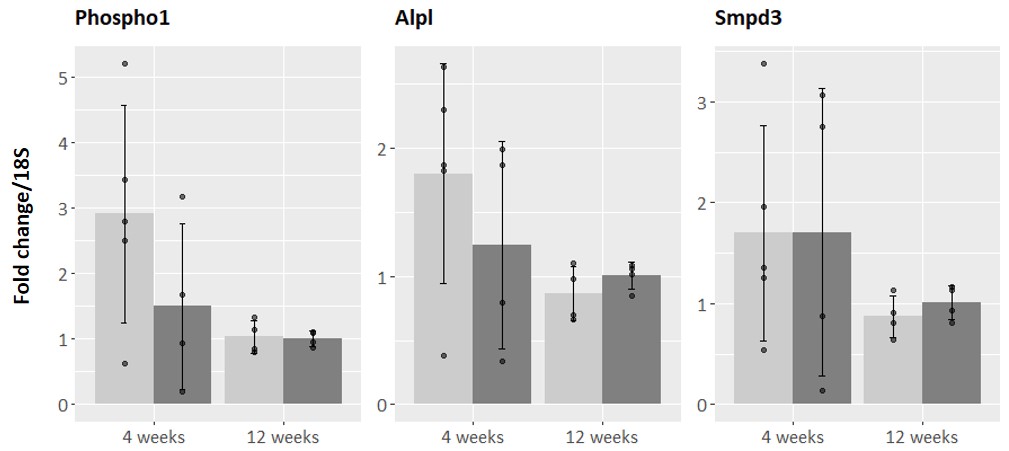

Supplement: Supplementary file 2 — Supplementary Figure S2 Micro‐computed tomography whole‐bone cortical analysis of moments of inertia in (A,B) 4‐week and (C,D) 12‐week tibias measured from 15% and 10% respectively to 90% bone length. Solid lines represent the mean ± standard deviation. Individual data points are plotted within each % length bracket. The results of statistical significance testing between genotypes performed using multiple t‐tests with Bonferroni correction for multiple comparisons are reported at each length bracket using colored bars. n = 4‐week Enpp6 −/− 4; 4‐week Enpp6 +/+ 5; 12‐week Enpp6 −/− 5; Enpp6 +/+ 4. [file JBM4-5-e10439-s002.jpg]
