# Supplementary material for: Ablation of Enpp6 Results in Transient Bone Hypomineralization
Source: JBMR Plus. 2020 Dec 8;5(2):e10439. doi: 10.1002/jbm4.10439 (PMC7872340; doi:10.1002/jbm4.10439)
Supplement: Supplementary file 3 — Supplementary Table S1 Oligonucleotide primers used in quantitative polymerase chain reaction experiments. [file JBM4-5-e10439-s003.docx]

**Supplementary Table 1.** Oligonucleotide primers used in quantitative polymerase chain reaction experiments.

| **Gene** | **Primer** | **Sequence** |
| --- | --- | --- |
| *Enpp6* | Forward | GTAGTCATCTTGGACCCTCTCATACTG |
|  | Reverse | GTGTGAGCTCTTACATGTGGACAGA |
| *Phospho1* | Forward | TTCTCATTTCGGATGCCAACA |
|  | Reverse | TGAGGATGCGGCGGAATA |
| *Alpl* | Forward | GGGACGAATCTCAGGGTACA |
|  | Reverse | AGTAACTGGGGTCTCTCTCTTT |
| *Smpd3* | Forward | CCCTCATCTTCCCATGTTACTGG |
|  | Reverse | GGCGCTTCTCATAGGTGGTG |
